# Synthetic Ligands of Cannabinoid Receptors Affect Dauer Formation in the Nematode *Caenorhabditis elegans*

**DOI:** 10.1534/g3.116.026997

**Published:** 2016-04-13

**Authors:** Pedro Reis Rodrigues, Tiffany K. Kaul, Jo-Hao Ho, Mark Lucanic, Kristopher Burkewitz, William B. Mair, Jason M. Held, Laura M. Bohn, Matthew S. Gill

**Affiliations:** * Department of Metabolism and Aging, The Scripps Research Institute, Jupiter, Florida 33458; † Department of Molecular Therapeutics, The Scripps Research Institute, Jupiter, Florida 33458; ‡ The Buck Institute for Research on Aging, Novato, California 94945; § Department of Genetics and Complex Diseases, School of Public Health, Harvard University, Boston, Massachusetts 02115; ** Division of Oncology, Washington University School of Medicine, St. Louis, Missouri 63110; †† Department of Anesthesiology, Washington University School of Medicine, St. Louis, Missouri 63110

**Keywords:** *C. elegans*, dauer, synthetic cannabinoids

## Abstract

Under adverse environmental conditions the nematode *Caenorhabditis elegans* can enter an alternate developmental stage called the dauer larva. To identify lipophilic signaling molecules that influence this process, we screened a library of bioactive lipids and found that AM251, an antagonist of the human cannabinoid (CB) receptor, suppresses dauer entry in *daf-2* insulin receptor mutants. AM251 acted synergistically with glucose supplementation indicating that the metabolic status of the animal influenced the activity of this compound. Similarly, loss of function mutations in the energy-sensing AMP-activated kinase subunit, *aak-2*, enhanced the dauer-suppressing effects of AM251, while constitutive activation of *aak-2* in neurons was sufficient to inhibit AM251 activity. Chemical epistasis experiments indicated that AM251 acts via G-protein signaling and requires the TGF-β ligand DAF-7, the insulin peptides DAF-28 and INS-6, and a functional ASI neuron to promote reproductive growth. AM251 also required the presence of the SER-5 serotonin receptor, but *in vitro* experiments suggest that this may not be via a direct interaction. Interestingly, we found that other antagonists of mammalian CB receptors also suppress dauer entry, while the nonselective CB receptor agonist, O-2545, not only inhibited the activity of AM251, but also was able to promote dauer entry when administered alone. Since worms do not have obvious orthologs of CB receptors, the effects of synthetic CBs on neuroendocrine signaling in *C. elegans* are likely to be mediated via another, as yet unknown, receptor mechanism. However, we cannot exclude the existence of a noncanonical CB receptor in *C. elegans*.

During development, *Caenorhabditis elegans* can enter an alternate larval stage, called the dauer larva, that allows the animal to survive adverse environmental conditions, such as high temperature, low food availability, and high population density (Golden and Riddle 1984a). Genetic analysis of dauer formation has defined neuroendocrine signals that act through a TGF-β-like signaling and an insulin-like signaling pathway, which converge on the cytochrome P450 DAF-9 and the nuclear receptor DAF-12 (Hu 2007). The identification of dafachronic acids (Motola *et al.* 2006) and other sterol acids (Held *et al.* 2006, Mahanti *et al.* 2014), which are produced by DAF-9 and act as ligands for DAF-12, confirmed the existence of lipophilic hormones that act downstream of TGF-β and insulin signaling to instruct the decision to proceed with reproductive growth. Likewise, the identification of ascarosides (ascr) as the bioactive components of dauer-inducing pheromone (Ludewig and Schroeder 2013) has confirmed the existence of small molecules that influence the activity of the neuroendocrine pathways in response to environmental conditions.

Evidence for small molecule signals that act through G protein-coupled receptors (GPCRs) to modulate dauer formation initially came from genetic evidence. *daf-11*, a guanylyl cyclase expressed in a subset of neurons, has a strong dauer constitutive (Daf-c) phenotype and was implicated in second messenger signaling downstream of chemosensory signaling and G-proteins (Birnby *et al.* 2000, Bargmann 2006). Furthermore, mutations in a subset of the nematode G protein subunits confer insensitivity to dauer pheromone (Lans and Jansen 2007, Zwaal *et al.* 1997, Lewis and Hodgkin 1977). The identification of ascarosides as the bioactive components of dauer pheromone further confirmed the role of GPCR signaling in modulating dauer entry. Two GPCRs, SRBC-64 and SRBC-66, have been shown to bind both ascr#2 and ascr#3 to induce dauer formation and likely function upstream of TGF-β and insulin-like signaling (IIS) (Kim *et al.*, 2009). Two other GPCRs, DAF-37 and DAF-38, have been shown to bind ascr#2 to promote dauer formation by repressing TGF-β signaling (Park *et al.* 2012). Likewise, *srg-36* and *srg-37* are GPCRs that are expressed in the sensory cilia of ASI neurons and mutations in these genes confer resistance to ascr#5, suggesting that they might bind this molecule to induce dauer formation (O’Rourke *et al.* 2013).

While ascaroside signaling is concerned with promoting dauer entry, food signals from the environment promote reproductive growth (Golden and Riddle 1984b). In contrast to pheromone signaling, much less is known about the identity of environmental food signals and their signaling mechanisms. We have previously determined that bacterial fatty acids can promote recovery from the dauer stage via a mechanism that is dependent on *daf-11* (Kaul *et al.* 2014), suggesting that food signals may also mediate their effects via GPCR signaling. In addition to small molecules that are sensed from the environment, it is likely that other endogenous small molecules contribute to the decision to proceed with reproductive growth or enter dauer. Indeed, we have previously found that a molecule from the N-acyl ethanolamine class of bioactive lipids can promote reproductive growth under dauer-inducing conditions (Lucanic *et al.* 2011). The existence of ∼80 cytochrome P450s (Menzel *et al.* 2001), which have the potential to synthesize small molecules, along with 284 nuclear receptors (Gissendanner *et al.* 2004, Robinson-Rechavi *et al.* 2005, Van Gilst *et al.* 2002), as well as over 1000 predicted G-protein coupled receptors in the *C. elegans* genome (Keating *et al.* 2003, Robertson and Thomas 2006), indicates that there are likely to be many more pathways that are responsive to small molecules in the worm. Despite this, we still know very little about the identity of other lipophilic hormones or small molecules that affect dauer formation or other phenotypes in the worm.

In this study, we took a screening approach, using a small library of bioactive lipids and related compounds, to identify small molecules that could promote reproductive growth in Daf-c mutants. We found that AM251, an inverse agonist/antagonist of the mammalian CB receptor (Gatley *et al.* 1996), suppressed dauer formation in *daf-2* insulin receptor mutants, by acting through G-protein signaling to activate TGF-β and insulin peptide pathways in the ASI chemosensory neuron. Since *C. elegans* does not possess obvious orthologs of the mammalian CB receptors (McPartland 2004, McPartland *et al.* 2006), AM251 likely acts via a different receptor mechanism. However, we also observed that a number of other synthetic CBs, both antagonists and agonists, also affect dauer formation, raising the possibility that the worm expresses a novel CB-like receptor that has conserved function but sequence divergence compared with the canonical mammalian CB receptors.

## Materials and Methods

### Chemicals

AM251, SR141716A (Rimonabant), URB447, LH21, AM630, O-2545, and CP55,940 were obtained from Cayman Chemical (Ann Arbor, MI) and Gp1a was obtained from Tocris Bioscience (Minneapolis, MN).

### C. elegans maintenance and strains

*C. elegans* strains were maintained as previously described (Brenner 1974). The following strains were obtained from the *Caenorhabditis* Genetics Center at the University of Minnesota: Bristol N2 (wild type), DR1572[*daf-2(e1368)*], DR1568[*daf-2(e1371)*], RB653[*ogt-1(ok430)*], CB1372[*daf-7(e1372)*], CB1364[*daf-4(e1364)*], DR47[*daf-11(m47)*], RB754[*aak-2(ok524)*], CB928[*unc-31(e928)*], NL332[*gpa-1(pk15)*], NL335[*gpa-3(pk35)*], NL790[*gpa-4(pk381)*], NL1137[*gpa-5(pk376)*], NL1146[*gpa-6(pk480)*], NL1147[*gpa-10(pk362)*], NL788[*gpa-14(pk342)*], RB2277[*ser-5(ok3087)*], MT15434[*tph-1(mg280)*], *PY6560* [*srbc-64(tm1946)*], and *PY6523*[*srbc-66(tm2943)*]. *aak-2(ok524)* was backcrossed to wild type N2 five times. Constitutively active (CA) AMPK strains—global expression - *uthIs248[aak-2p*::*aak-2 genomic (aa1-321)*::*GFP*::*unc-54 3′UTR]*; neuronal expression - *wbmEx66(rab-3p*::*aak-2 (aa1-321 cDNA)*::*tdTomato*::*unc-54 3′UTR)*; intestinal expression - *wbmEx67(gly-19p*::*aak-2 (aa1-321 cDNA)*::*tdTomato*::*unc-54 3′UTR)*; muscle expression - *wbmEx68(myo-3p*::*aak-2 (aa1-321 cDNA)*::*tdTomato*::*unc-54 3′UTR*)—were generated as previously described (Burkewitz *et al.* 2015). *ins-6(tm2416)* and *daf-28(tm2308)* were obtained from Dr. Shohei Mitani at the National Bioresource Project at Tokyo Women’s Medical University School of Medicine and were backcrossed five times to N2.

The *ins-6* and *daf-28* mutants were each crossed into *daf-2(e1371)* as previously described (Kaul *et al.* 2014). All other double mutants were generated by standard methods and crosses were confirmed by PCR genotyping or sequencing where applicable.

The ASJ ablation strains *jxEx18[unc-122p*::*GFP]* and *daf-2(e1368)*; *jxEx100[pQZ37(trx-1p*::*ICE)*; *unc-122p*::*GFP]* were a kind gift from Dr. Joy Alcedo (Cornils *et al.* 2011). For ablation of the ASI neuron, 2 kb of the *gpa-4* promoter was inserted upstream of human caspase in the pV32 plasmid (a kind gift from V. Maricq) using *Pst*I and *Kpn*I restriction sites. To generate transgenic animals, *daf-2(e1368)* worms were injected with pPV32 *gpa-4p*::*ICE* (30ng/µl) and a *myo-2p*::*GFP* coinjection marker (5 ng/µl), and a *daf-2(e1368);jluEx122(gpa-4p*::*ICE*; *myo-2p*::*GFP)* stable line was identified through *myo-2p*::*GFP* expression.

### Dauer assays

Dauer assays were performed as previously described (Held *et al.* 2006). Compounds were resuspended in DMSO to a final concentration of 20 mM. For dose range experiments, serial dilutions were made to yield 10, 5, 2, 1, and 0.5 mM. 15 µL of each working solution of compound were added to 135 µL of water before being spotted onto a 4 mL NGM plate. Equal distribution of the compound throughout the agar was assumed to yield final concentrations of 50, 25, 10, 5, and 2.5 µM.

For the dauer shift assay, eggs from a synchronous lay were transferred to plates containing DMSO and placed at 25°. At different time intervals after the lay, worms were removed from the incubator and transferred to plates containing AM251 and returned to the incubator as quickly as possible. Two separate lays were performed, one in the morning and one in the evening such that one lay would yield the 24 hr and 30 hr time points and the other lay would yield the 12 hr and 18 hr time points. For glucose assays, glucose was added to the molten NGM prior to pouring plates and dauer assays performed as described above.

### SER-5/β-arrestin2 recruitment assay

*C. elegans ser-5* cDNA was amplified with a 5′ *Xho*I and a 3′ *Kpn*I site and cloned into pCMV HA-N (Clontech). The β-arrestin2 recruitment assay was performed as previously described (Barak *et al.* 1997, Johnson *et al.* 2003). Briefly, HEK cells were transfected with HA-*ser-5* (5 μg) and mouse β-arrestin2-EGFP (2 μg) using the Gene Pulser Xcell electroporation system (Bio-Rad). After transfection, cells were plated on collagen-coated glass-bottom dishes and incubated overnight. Cells were then serum starved in Opti-MEM media without phenol red for 60 min. An Olympus FluoView 1000 confocal microscope was used to image β-arrestin2-EGFP translocation in live cells. Single focal plane images were captured using 100X objective after serum starvation for basal activity, and between 5 and 60 min after drug treatment. To ensure cells that were not stimulated by AM251 were able to respond to drug treatment, cells were treated with 10 μM serotonin after AM251 treatment.

### Statistical analysis

The percentages of dauers and nondauers were calculated for each trial, and trials set up on different days were deemed to be biological replicates. Statistical analysis was performed using GraphPad Prism. Data are presented as mean + sd and were analyzed by Student’s *t*-test for k = 2 groups or one-way ANOVA for k ≥ 3 groups with Sidak’s multiple comparisons test for pairwise comparisons.

### Data availability

All strains and vectors generated are available upon request. All data necessary for confirming the conclusions presented in the article are available in Supplementary Material, File S1.

## Results

### The CB receptor antagonist AM251 suppresses dauer formation in C. elegans

**Figure 1:**
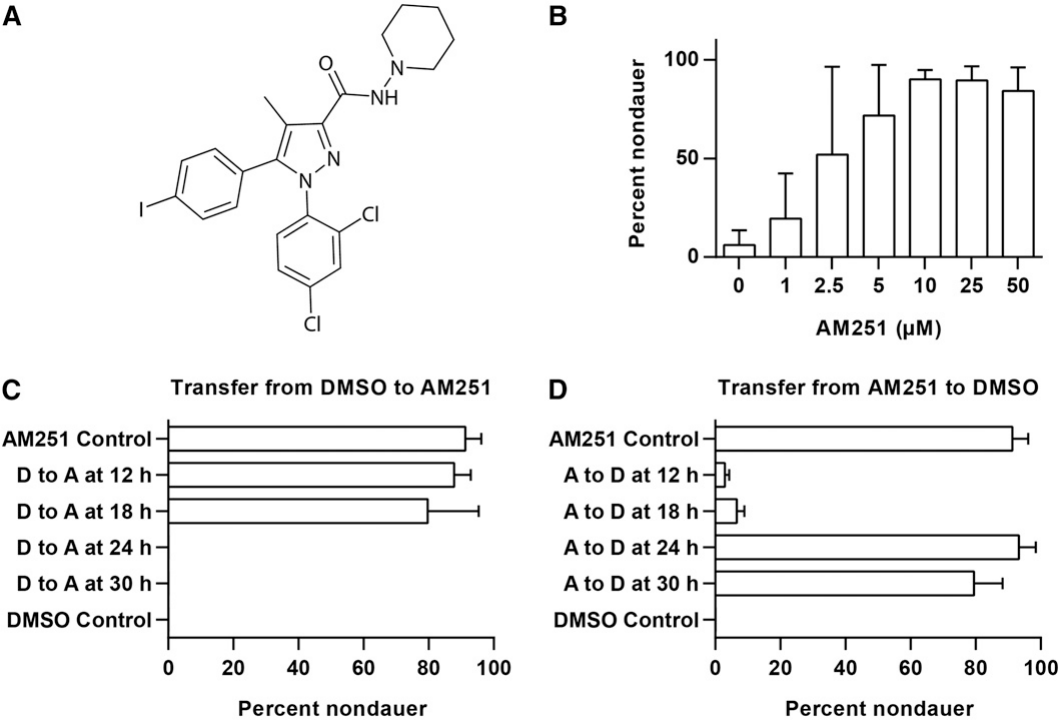
The cannabinoid (CB) receptor antagonist AM251 suppresses dauer formation. (A) Structure of the CB1 receptor antagonist/inverse agonist AM251. (B) AM251 promotes reproductive growth in *daf-2(e1371)* mutants at 25° in a dose-dependent manner. (C) *daf-2(e1368)* worms raised on DMSO (D, dimethyl sulfoxide) and transferred to AM251 (A) up to 24 hr after the egg lay develop into reproductive adults, while worms transferred at 24 hr, or later, are committed to dauer formation and AM251 has no effect. (D) *daf-2(e1368)* worms raised on AM251 (A) and transferred to DMSO (D) up to 24 hr after the egg lay develop into dauers while worms maintained on AM251 for 24 hr or 30 hr and then transferred are committed to reproductive growth and the absence of AM251 has no effect.

To identify lipid signaling molecules that suppress dauer entry in *C. elegans*, we screened the Bioactive Lipid Library (Enzo Life Sciences) in a *daf-2(e1371)* mutant background at 25°. Of the 204 compounds screened, only one compound, AM251 (Figure 1A), robustly promoted growth of *daf*-2 mutants to the gravid adult stage under dauer-inducing conditions. To confirm the activity of AM251, we carried out dose response experiments and found that this molecule reproducibly promoted growth of *daf-2* mutants to adulthood in a dose-dependent manner, with 5 µM AM251 sufficient to rescue almost 100% of animals (Figure 1B).

### AM251 acts early in development to promote reproductive growth

To examine the timing requirement for AM251, *daf-2(e1368)* animals from a synchronous egg lay were raised at 25° on plates containing DMSO vehicle (dauer-inducing conditions) and transferred to plates containing AM251 at different time points. Worms that were transferred to AM251 at 12 hr or 18 hr after the egg lay developed into adult, nondauer animals in the same way as the AM251 control (Figure 1C). However, worms that were transferred from DMSO to AM251 at 24 hr and 30 hr after the egg lay continued to develop into dauers, as did animals that were only exposed to DMSO. Conversely, worms raised on AM251 and shifted to DMSO at 12 hr or 18 hr after the egg lay developed into dauers in the same way as the DMSO control (Figure 1D), but worms that were transferred from AM251 to DMSO at 24 hr and 30 hr after the egg lay developed into reproductive adults, as did animals that were only exposed to AM251. These data show that AM251 exposure in late L1/early L2 is required and sufficient to promote reproductive growth. In addition, the failure of AM251 to promote reproductive growth in animals shifted after 24 hr suggests that it cannot alter the fate of L2d/predauer animals.

### AM251 activity is augmented by glucose supplementation

**Figure 2:**
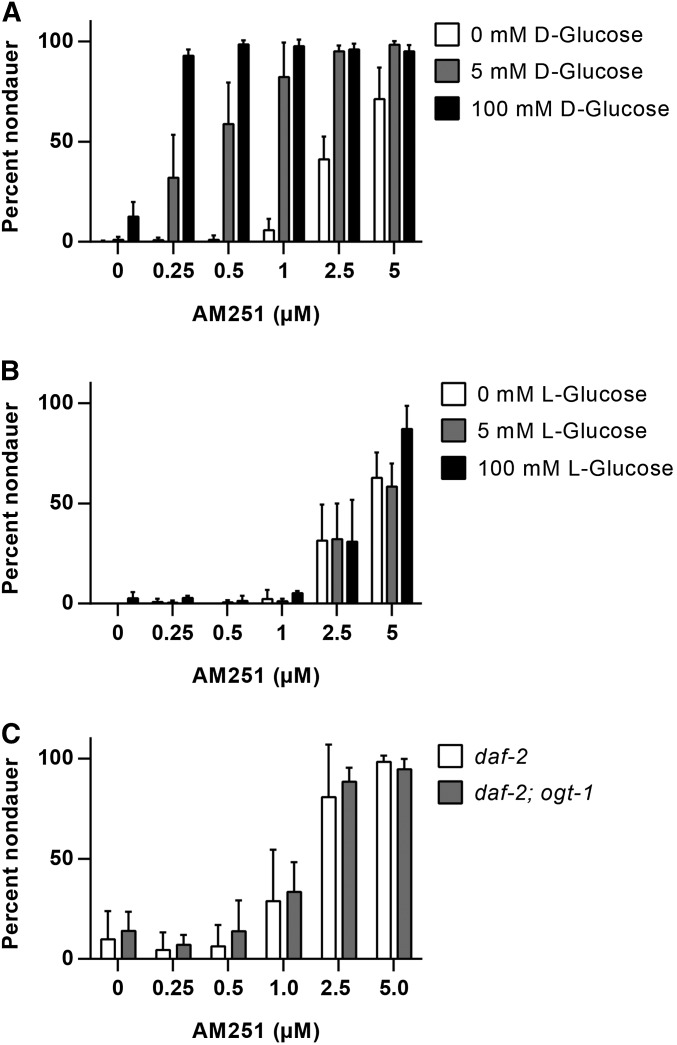
AM251 activity is augmented by glucose supplementation. (A) The ability of AM251 to promote reproductive growth in *daf-2(e1368)* mutants at 25° is augmented by D-glucose. Pairwise comparisons for 0 mM glucose *vs.* 5 mM glucose and 100 mM glucose at each dose of AM251 all had *P* < 0.001, except at 0 µM AM251 where 0 mM glucose *vs.* 100 mM glucose had *P* < 0.01. (B) L-Glucose supplementation does not enhance the ability of AM251 to promote reproductive growth in *daf-2(e1368)* mutants. (C) Deletion of the O-GlcNac (O-linked-N-acetylglucosamine) transferase, *ogt-1(ok430)*, has no effect on the ability of AM251 to promote reproductive growth in *daf-2(e1368)*.

Supplementation with glucose has been shown to rescue the Daf-c phenotype of *daf-2* mutants at semipermissive temperatures (Lee *et al.* 2009, Mondoux *et al.* 2011), via a mechanism that involves O-linked-N-acetylglucosamine (O-GlcNac) cycling (Forsythe *et al.* 2006, Hanover *et al.* 2005, Lee *et al.* 2010). We therefore asked whether AM251 promotes reproductive growth via a similar mechanism. At the restrictive temperature of 25°, exposure to 100 mM D-glucose promoted reproductive growth in only 10% of animals (Figure 2A). Under these conditions, 5 µM AM251 alone resulted in approximately 70% rescue, with lower doses showing very little effect (Figure 2A). However, combined exposure to glucose and AM251 resulted in a dose-dependent, synergistic increase in the number of nondauer animals (Figure 2A). This effect required metabolism of glucose, since in the presence of L-glucose, which cannot be metabolized, there was no additional effect over that of AM251 alone (Figure 2B). Loss of function mutations in the O-GlcNac transferase *ogt-1* lead to decreased O-GlcNacylation of proteins and diminished the ability of glucose to suppress dauer formation in *daf-2* mutants (Mondoux *et al.* 2011). However, AM251 was still able to rescue dauer formation in a *daf-2*; *ogt-1* mutant background (Figure 2C). Collectively, these data suggest that AM251 and glucose promote reproductive growth via parallel mechanisms, and also indicate that the activity of AM251 is influenced by the metabolic status of the animal.

### Neuronal AMPK activity inhibits the effects of AM251

**Figure 3:**
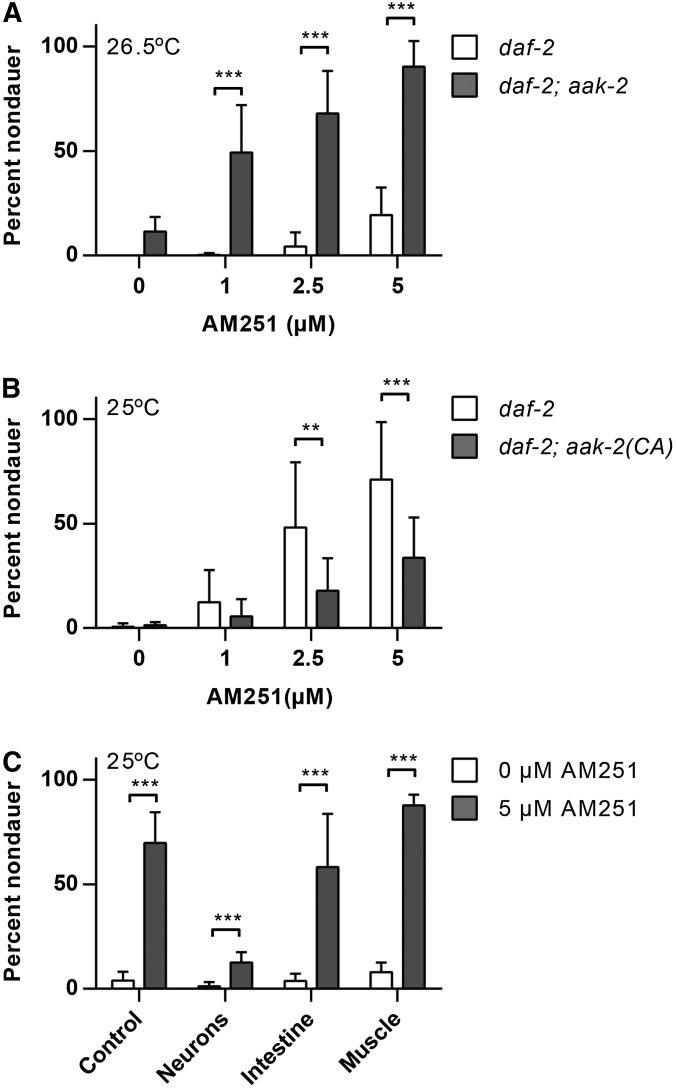
AM251 is inhibited by neuronal AMPK activity. (A) AM251 suppresses dauer formation in *daf-2(e1368)*; *aak-2(ok524)* mutants at 26.5°. (B) The ability of AM251 to suppress dauer formation in *daf-2(e1368)* mutants is inhibited by the presence of constitutively active AAK-2 (*daf-2(e1368)*; *aak-2(CA)*). (C) Constitutively active (CA) AAK-2 in neurons, but not in intestine or muscle, inhibits the ability of AM251 to promote reproductive growth in *daf-2(e1368)* at 25°. For all panels, pairwise comparisons are indicated: ** *P* < 0.01 *** *P* < 0.001. AAK, AMP-activated kinase subunit; AMPK, AMP regulated kinase.

The AMP regulated kinase, AMPK, is an important sensor of cellular energy levels and an increase in the AMP to ATP ratio arising from low nutrition results in phosphorylation and activation of AMPK (Hardie 2011). In *C. elegans*, loss of function mutations in one of the AMPK α subunits, *aak-2*, behave like AM251 treatment and suppress dauer entry in *daf-2* mutants at 25° (Apfeld *et al.* 2004). Thus, we reasoned that if AM251 was inhibiting AMPK activity, there should be no further suppression of the Daf-c phenotype in *daf-2*; *aak-2* mutants treated with AM251. Contrary to this, we found that AM251 was fully effective in promoting reproductive growth in *daf-2(e1368)*; *aak-2(ok524)* double mutants at 26.5° (Figure 3A). This suggests that AM251 is not acting via inhibition of AAK-2, but rather that AAK-2 activity may limit the dauer rescuing activity of this molecule.

If wild-type AMPK activity limits the effectiveness of AM251, we reasoned that constitutively active AAK-2 should further inhibit the ability of AM251 to suppress dauer formation. We therefore introduced a constitutively active version of AMPK, *aak-2(CA)* (Mair *et al.* 2011), into the *daf-2* mutant background and asked whether AM251 was still able to suppress dauer formation. There was no increase in the basal level of dauer formation in the *daf-2*; *aak-2(CA)* animals, and while they did respond to AM251 in a dose-dependent manner, there was a significant reduction in the magnitude of the response compared with *daf-2* controls (Figure 3B). These data show that constitutive activation of AMPK inhibits the ability of AM251 to suppress dauer formation by a parallel mechanism.

To determine where AAK-2(CA) acts to inhibit AM251, we expressed it in different tissues and examined the ability of AM251 to suppress dauer entry in *daf-2* mutants. AM251 was still able to suppress dauer formation in *daf-2* mutants with activated AMPK in either the intestine or in body wall muscle, but was unable to promote reproductive growth to the same extent when AMPK was activated in neurons (Figure 3C). These data indicate that AAK-2 activity in neuronal tissues antagonizes the growth promoting activity of AM251 and are consistent with AM251 acting through a neuronal mechanism to inhibit dauer formation.

### AM251 acts via TGF-β signaling and insulin peptides in the ASI neuron

**Figure 4:**
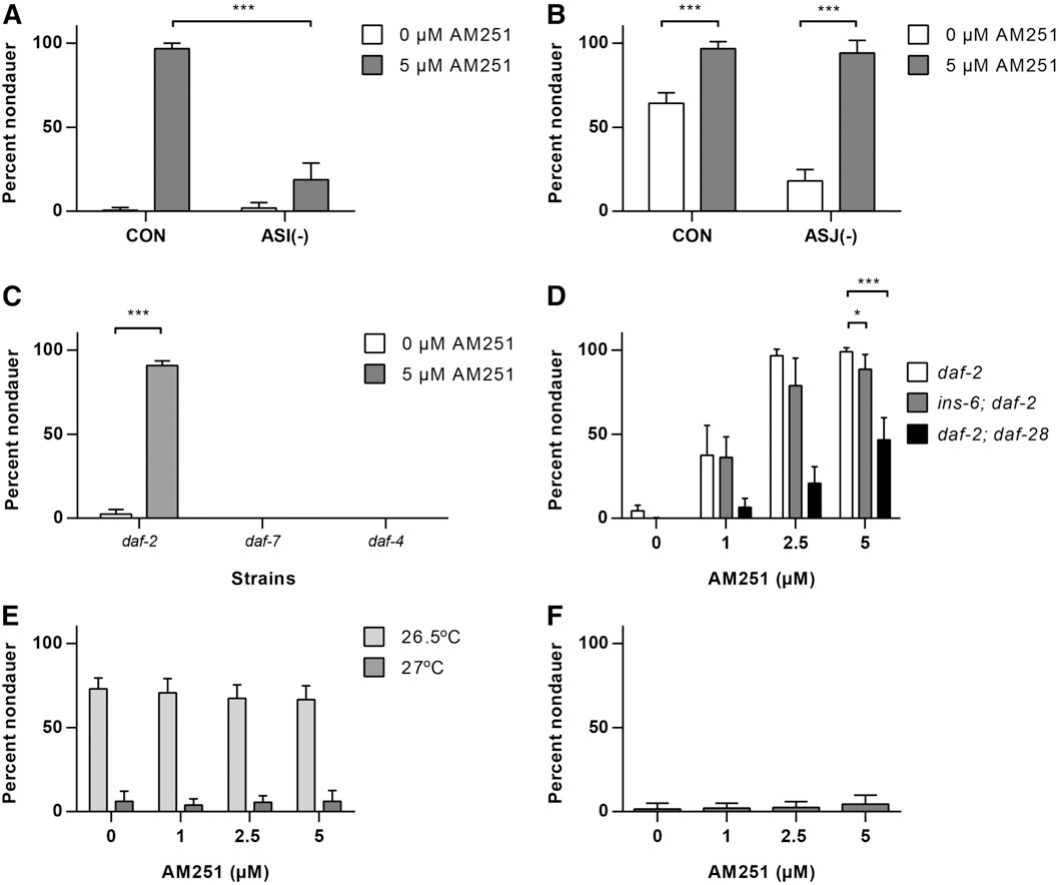
AM251 requires both TGF-β and insulin secretion from the ASI neuron. (A) Genetic ablation of the ASI sensory neuron blocks the ability of AM251 to suppress dauer formation in the *daf-2(e1368)* mutant background at 25°. (B) Genetic ablation of the ASJ sensory neuron has no effect on the ability of AM251 to suppress dauer formation in the *daf-2(e1368)* mutant background at 25°. (C) 5 µM AM251 promotes reproductive growth in *daf-2(1368)* mutants, but not in *daf-7(e1372)* or *daf-4(e1364)* mutants. (D) AM251 rescues the dauer formation phenotype of *ins-6(tm2416)*; *daf-2(e1371)* and *daf-2(e1371)*; *daf-28(tm2308)* double mutants at 25°. (E) AM251 does not rescue the dauer formation phenotype of an *ins-6(tm2416)*; *daf-28(tm2308)* double mutant at 26.5° or 27°. ANOVA by dose: 26.5° *P* = ns, 27° *P* = ns. (F) AM251 does not suppress the Daf-c phenotype of *unc-31(e928)* mutants at 27°. ANOVA by dose *P* = ns. For all panels, pairwise comparisons are indicated: * *P* < 0.05, *** *P* < 0.001. CON, control; ns, not significant.

Laser ablation studies have identified a number of sensory neurons that are involved in dauer entry and exit (Bargmann and Horvitz 1991). Of these, the ASI neuron plays a major role in promoting reproductive growth and inhibiting dauer entry, with a lesser role for the ASJ neuron (Bargmann and Horvitz 1991), and thus ASI is a strong candidate for mediating the effects of AM251. Therefore, we examined dauer entry in response to AM251 in *daf-2* mutants in which either the ASI or ASJ neuron was ablated through tissue specific expression of a cytotoxic caspase (Cornils *et al.* 2011). Ablation of the ASI neuron strongly reduced the ability of AM251 to promote reproductive growth (Figure 4A), but loss of the ASJ neuron had no effect (Figure 4B). These data show that the effect of AM251 in suppressing dauer entry is mediated via the ASI neuron.

The ASI neuron promotes reproductive growth via the secretion of the TGF-β ligand DAF-7 (Ren *et al.* 1996), as well as insulin peptides, such as DAF-28 (Li *et al.* 2003) and INS-6 (Cornils *et al.* 2011). We therefore tested if AM251 was acting through these pathways to promote reproductive growth. We found that AM251 did not rescue dauer formation in *daf-7* mutants, which lack a TGF-β ligand, nor did it have any effect on *daf-4* mutants, which have a defective TGF-β receptor (Figure 4C). These data show that AM251 requires a functional TGF-β signaling pathway for its effect on dauer formation. We also tested the requirement for *daf-28* and *ins-6* because they are expressed early in larval development (Baugh *et al.* 2011) and both have previously been implicated in dauer formation (Li *et al.* 2003, Cornils *et al.* 2011). AM251 was able to suppress dauer formation in *ins-6(tm2416)*; *daf-2(e1371)* and *daf-2(e1371)*; *daf-28(tm2308)* double mutants at 25°, although the response was significantly reduced compared with *daf-2* alone and was more pronounced in the *daf-2*; *daf-28* double mutant (Figure 4D). These data are consistent with the assertion that *daf-28* is more important than *ins-6* in promoting reproductive growth (Cornils *et al.* 2011), but suggest that AM251 influences secretion of both insulins. AM251 also failed to promote reproductive growth in an *ins-6(tm2416);daf-2(e1371);daf-28(tm2308)* triple mutant, but we were concerned that the strong Daf-c phenotype (∼95% dauers at 15°) would obscure the interpretation of dependency (data not shown). We therefore examined whether AM251 could prevent dauer formation in an *ins-6*; *daf-28* double mutant in the presence of a wild-type *daf-2* receptor. *ins-6(tm2416)*; *daf-28(tm2308)* double mutants are Daf-c at 27° (Cornils *et al.* 2011), and we found that AM251 was unable to rescue the Daf-c phenotype of the double mutant at either fully restrictive or semipermissive temperatures (Figure 4E). This result suggests that insulin peptide secretion after exposure to AM251 is required for promoting reproductive growth. To further test this, we evaluated the effect of AM251 in *unc-31* mutants that lack a calcium activated protein for secretion (CAPS) homolog that is required for DAF-7 and insulin secretion via dense core vesicle docking (DCV) (Ailion *et al.* 1999, Speese *et al.* 2007). Consistent with a neurosecretory mechanism, we found that AM251 had no effect on the Daf-c phenotype of *unc-31* mutants at 27° (Figure 4F).

### G-protein signaling mediates the effects of AM251 on dauer formation

**Figure 5:**
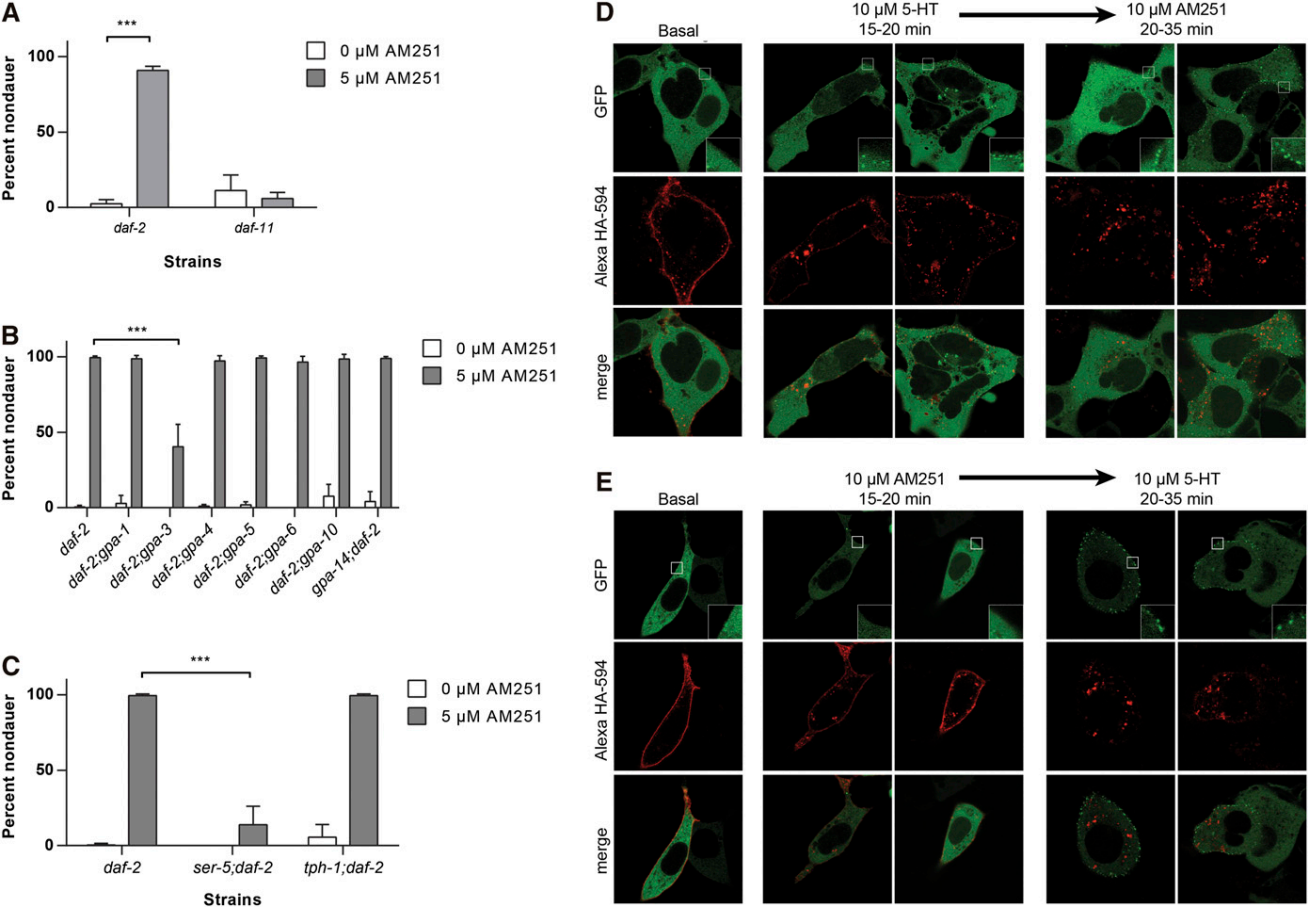
AM251 functions upstream of *gpa-3* and *ser-5* to suppress dauer formation. (A) AM251 does not prevent dauer formation in *daf-11(m47)* mutants. (B) The *gpa-3* G-protein subunit is partially required for AM251 to prevent dauer formation in *daf-2(e1368)* mutants. (C) AM251 requires the serotonin receptor *ser-5* to prevent dauer formation in the *daf-2(e1368)* background, but is not affected by the loss of *tph-1*. (D) Heterologous expression of HA-tagged SER-5 and mouse β-arrestin2-EGFP in HEK 293T cells. Incubation with 10 μM 5-HT for 20–35 min robustly induced β-arrestin2-EGFP (green punctae) recruitment to cell surface membranes of HEK-293T cells expressing HA-SER-5 (red). As is typical for GPCRs including mammalian serotonin receptors, internalization of the receptors can also be detected following serotonin treatment (intracellular red punctae). Addition of AM251 did not reverse serotonin-induced β-arrestin2-EGFP translocation or receptor internalization, suggesting that it is not acting as an antagonist at the receptor. (E) In contrast, β-arrestin2-EGFP recruitment was not observed in cells treated with 10 μM AM-251 for 20–35 min and this treatment failed to prevent 10 μM 5-HT induced β-arrestin2-EGFP recruitment and HA-SER-5 internalization, again, suggesting that AM251 does not act as an antagonist at this receptor. The experiment was performed over three individual transfections of HEK-293T cells that received AM251 and serotonin each time. Images are representative of over 50 images collected for each treatment. For all panels, pairwise comparisons are indicated: *** *P* < 0.001. EGFP, enhanced green fluorescent protein; GPCR, G protein-coupled receptor.

The guanylyl cyclase DAF-11 is expressed in the ASI neuron and has been shown to be required for both TGF-β and insulin peptide expression (Li *et al.* 2003, Murakami *et al.* 2001). AM251 was not able to promote reproductive growth in *daf-11* mutants (Figure 5A), suggesting that it requires functional DAF-11 to promote secretion of neuropeptides from the ASI neuron. Since *daf-11* is hypothesized to work downstream of GPCR signaling (Birnby *et al.* 2000, Bargmann 2006), we tested if G-protein subunits are required for the effects of AM251. We focused on those G-proteins that are expressed in the ASI neuron, and crossed mutants into the *daf-2(e1368)* background to evaluate their effects on the ability of AM251 to suppress dauer formation. Of the seven G-proteins tested, we found that only *gpa-3* is partially required for the effects of AM251 (Figure 5B). This indicates that *gpa-3* is functioning upstream of TGF-β and insulin secretion and impacts the ability of AM251 to promote reproductive growth.

### AM251 requires the serotonin receptor ser-5 to suppress dauer formation

As *C. elegans* lacks orthologs of CB receptors (McPartland 2004, McPartland *et al.* 2006), it is likely that AM251 acts through another receptor type to influence dauer formation. We considered the serotonin receptor ortholog *ser-5* to be a strong candidate, as a previous study had shown that *ser-5* influences dauer exit by modulating AMPK activity and dense core vesicle secretion (Cunningham *et al.* 2014). Consistent with a requirement for *ser-5*, we found that AM251 was unable to promote reproductive growth in *ser-5;daf-2* double mutants (Figure 5C). Interestingly, we found that AM251 was able to fully rescue dauer formation in *tph-1;daf-2* double mutants, which lack the ability to synthesize serotonin (Figure 5C). This suggests that AM251 is not acting through SER-5 by simply modulating the availability of its ligand.

To directly test whether AM251 had functional activity at SER-5, we employed a β-arrestin2 translocation assay in HEK-293T cells (Barak *et al.* 1997, Johnson *et al.* 2003). We coexpressed HA-tagged SER-5 and mouse β-arrestin2 fused to EGFP, in order to ask if AM251 was able to elicit β-arrestin2 recruitment to the cell surface. As a positive control we treated cells with 10 μM 5-HT, and observed the formation of β-arrestin2-EGFP puncta close to the cell membrane, demonstrating that 5-HT is an agonist of SER-5 (Figure 5, D and E). In addition, HA staining of HA-SER-5 transfected cells revealed an increase in internalized SER-5 after serotonin treatment providing further evidence that 5-HT is an agonist at SER-5. Treatment with 10 μM AM251, however, had no effect on the localization of β-arrestin2-EGFP, indicating that AM251 is not an agonist at SER-5. Moreover, AM251 was not able to reverse 5-HT stimulated β-arrestin2-EGFP recruitment, nor was it able to block subsequent 5-HT activity, demonstrating that AM251 is also not an antagonist at SER-5. Together, these data suggest that AM251 does not have functional activity at SER-5.

### Multiple CB receptor ligands modulate dauer formation

**Figure 6:**
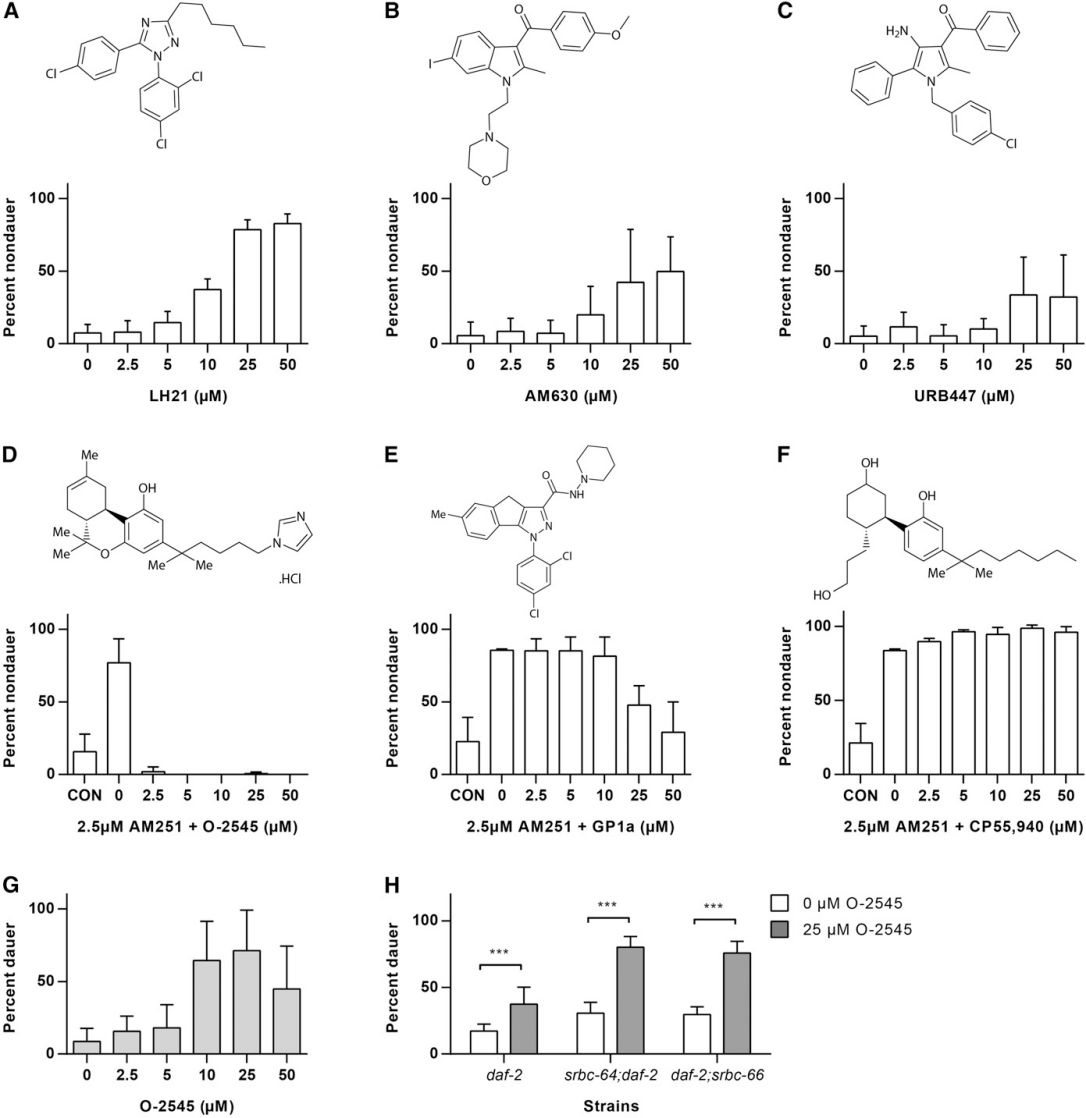
Other CB receptor agonists and antagonists influence dauer formation in *daf-2* mutants. (A–C) CB receptor antagonists promote reproductive growth in *daf-2(e1368)* at 25°: (A) LH21 is a silent CB1 antagonist. (B) AM630 is a selective CB2 antagonist. (C) URB447 is a mixed central CB1 receptor antagonist/peripheral CB2 agonist. (D) The growth-promoting effect of 2.5 µM AM251 in *daf-2(e1368)* is inhibited by the presence of the nonselective CB1/CB2 agonist O-2545. (E) The growth-promoting effect of 2.5 µM AM251 in *daf-2(e1368)* is partially inhibited by the presence of the CB2 selective agonist GP1a. (F) The growth-promoting effect of 2.5 µM AM251 in *daf-2(e1368)* is not affected by the presence of the nonselective CB1/CB2 agonist CP55,940. (G) O-2545 alone promotes dauer formation in *daf-2(e1368)* at the semipermissive temperature of 23.6°. (H) Induction of dauer formation by 25 µM O-2545 in *daf-2(e1368)* at 23.6° is not affected by deletion of the ascaroside receptors *srbc-64* and *srbc-66*. Pairwise comparisons are indicated: *** *P* < 0.001. CB, cannabinoid; CON, control.

Although it is likely that AM251 acts through a non-CB receptor mechanism, it also remains a possibility that worms possess a functional ortholog of CB receptors. In support of this latter hypothesis, we found that a number of other CB receptor antagonists were capable of suppressing dauer formation in *daf-2* mutants. These include the silent CB1 antagonist LH21 (Jagerovic *et al.* 2004), the selective CB2 antagonist AM630 (Ross *et al.* 1999), as well as the mixed central CB1 receptor antagonist/peripheral CB2 agonist URB447 (LoVerme *et al.* 2009) (Figure 6, A–C). We also examined a number of CB receptor agonists for their ability to inhibit the actions of AM251 in suppressing dauer formation. Worms were exposed to increasing concentrations of the agonists in the presence of 2.5 µM AM251, a dose that is capable of rescuing dauer formation in the majority of animals. O-2545, a water soluble analog of the main psychoactive component of cannabis, Δ9-tetrahydrocannabinol, and a nonselective CB1/2 receptor agonist (Martin *et al.* 2006), was able to potently suppress AM251-mediated reproductive growth (Figure 6D). The CB2 receptor agonist GP1a (Murineddu *et al.* 2006) was also able to block the effects of AM251 to a lesser extent (Figure 6E), while another nonselective CB1/2 receptor agonist, CP55,940 (Pertwee 1999), had no effect (Figure 6F).

The ability of O-2545 to prevent the effects of AM251 led us to further examine whether it was able to induce dauer formation when administered alone. We found that at the semipermissive temperature of 23.6°, O-2545 did indeed induce a significant number of dauers at 10 µM and 25 µM, while at higher concentrations it resulted in a significant number of animals that were slow growing or arrested in the early larval stages (Figure 6G). The ability of O-2545 to induce dauer formation did not require *srbc-64* or *srbc-66*, suggesting that this CB receptor agonist was not acting via these ascaroside receptors (Figure 6H).

## Discussion

We screened a small library of bioactive lipids and identified the synthetic mammalian CB1 receptor inverse agonist/antagonist AM251 as a robust modifier of dauer formation in the worm. CB receptor antagonists were developed as an antiobesity therapy, with the hypothesis that inhibition of the CB receptor in the central nervous system would decrease feeding and thus lead to weight loss (Carai *et al.* 2005). One such compound is Rimonabant, a closely-related analog of AM251, which we also found to suppress dauer formation in worms, to the same extent as AM251 (data not shown). In clinical trials, the degree to which Rimonabant promoted weight loss and the extent of its beneficial effects on lipid profiles suggested that its mechanism went beyond the central inhibition of food intake (Scheen 2008). There is now evidence that CBs influence glucose homeostasis and insulin sensitivity, independently of their effects in the central nervous system (Juan-Pico *et al.* 2006, Nogueiras *et al.* 2009).

We have generated a number of lines of evidence that suggest that AM251 also elicits changes in insulin sensitivity in *C. elegans*, which leads to suppression of dauer entry. First, AM251 activity required the presence of the insulin peptides *daf-28* and *ins-6*, as well as *unc-31*, which encodes a protein that is required for dense core vesicle docking and neuropeptide secretion. The *daf-2(e1368)* and *daf-2(e1371)* mutations that we used in this study are hypomorphic and alter single amino acid residues in the extracellular, ligand binding domain of *daf-2* (Patel *et al.* 2008). Thus, it is likely that these mutants have altered affinity for insulin peptides, and, consequently, their temperature sensitive Daf-c phenotypes could be overcome by increases in insulin peptide availability. Although AM251 also required the TGF-β ligand *daf-7*, this could be a consequence of *daf-7* mutants being functionally deficient in insulin signaling, rather than a direct action of AM251 on TGF-β signaling, since *daf-7* mutations lead to down-regulation of many components of the insulin signaling pathway (Liu *et al.* 2004). Second, AM251 required an intact ASI neuron, but was not affected by ablation of the ASJ neuron. ASI is known to be important for promoting reproductive growth (Bargmann and Horvitz 1991) and expresses DAF-7, DAF-28, and INS-6 during development (Ren *et al.* 1996, Li *et al.* 2003, Cornils *et al.* 2011). Third, timing experiments indicated that AM251 exposure early in development was sufficient to promote reproductive growth. These data recapitulate the temperature shift experiments of Swanson and Riddle (Swanson and Riddle 1981), that originally defined the dauer decision window and indicate that AM251 acts in late L1/early L2. Importantly, this is also the time at which *daf-28* and *ins-6* expression peak during development (Baugh *et al.* 2011).

The synergistic interaction between AM251 and glucose in modifying dauer formation is also consistent with AM251 affecting insulin sensitivity. Glucose supplementation prevents dauer entry only under semipermissive conditions (Lee *et al.* 2009, Mondoux *et al.* 2011), and one interpretation of this is that under these conditions there is enough residual insulin signaling to generate ATP when more glucose becomes available. However, under fully restrictive conditions, insulin signaling drops below a threshold and metabolism is shifted away from energy utilization to energy storage (Braeckman *et al.* 2009). In this situation, the addition of supplemental glucose results in increased energy storage and has no effect in promoting reproductive growth. Thus, at low doses, where AM251 alone is not able to increase insulin signaling sufficiently to prevent dauer entry, the addition of glucose provides additional substrate to drive catabolism and energy production beyond a threshold that is sufficient to support reproductive growth.

Further evidence that AM251 activity is influenced by the metabolic status of the animal comes from its interaction with AMPK signaling. Loss of function mutations in the *aak-2* subunit behave like AM251 in that they suppress dauer formation in *daf-2* mutants (Apfeld *et al.* 2004). Although epistasis analysis indicated that AM251 does not require AMPK for its activity, we did find that constitutive activation of *aak-2* in neurons was able to block AM251 signaling. This suggests that the AM251 signaling cascade acts in parallel to energy sensing by AMPK. In mammals, AMPK activity influences insulin secretion from pancreatic β-cells in a number of ways, including glucose metabolism, K^+^/ATP channel trafficking, insulin granule docking, and insulin gene transcription (Fu *et al.* 2013). Thus, the effect of AMPK activity on AM251 function may be via its effects on insulin peptide transcription and/or insulin secretion, rather than directly inhibition of AM251 signaling pathways.

The dependence on *daf-7*, *ins-6*, and *daf-28* also support the idea that the ASI sensory neuron is a target tissue of AM251. However, whether this is mediated through a direct interaction with a receptor expressed on this neuron or indirectly by acting on another neuron, which in turn influences ASI activity, remains to be determined. AM251 also required the activity of the guanylyl cyclase DAF-11, which is expressed in a subset of amphid sensory neurons, including ASI (Birnby *et al.* 2000), and is thought to function downstream of chemosensory G-protein coupled receptors (Bargmann 2006). In the absence of CB receptor orthologs, we considered the serotonin receptor SER-5 to be a good candidate for an AM251 target, principally because SER-5 had been shown to act in the ASI neuron to influence dauer exit by modulating AMPK activity and dense core vesicle secretion (Cunningham *et al.* 2014). Genetic experiments indicated that *ser-5* was indeed required for the activity of AM251 but, intriguingly, AM251 did not require the activity of *tph-1*, which is required for serotonin synthesis (Sze *et al.* 2000). This suggests that AM251 is not acting to modulate serotonin availability at the SER-5 receptor and raised the possibility that it could directly interact with SER-5. This hypothesis, however, was not supported by molecular pharmacology studies. A β-arrestin2 translocation assay, failed to reveal functional affinity for AM251 and the worm SER-5 receptor. β-arrestin2 acts as a signaling scaffold for many GPCRs (Orgel 1963) and the translocation assay provides a means of determining whether a ligand acts through a target receptor without any knowledge of the subset of G-proteins that are required (Peters *et al.* 2012). Importantly, we were able to demonstrate that 5-HT treatment of cells transfected with HA-SER-5 does lead to β-arrestin2-EGFP being translocated to the cell surface and internalization of the receptor, providing direct evidence that 5-HT does activate SER-5. Unfortunately, we were not able to observe any effect of AM251 alone on β-arrestin2-EGP recruitment, nor did we see any evidence that AM251 could compete with 5-HT. Taken together, these data suggest that AM251 might be either functioning upstream of SER-5 by altering the levels of a non-5-HT-like ligand or, alternatively, that AM251 acts on a different receptor target that interacts with the SER-5 receptor. In this respect, it is noteworthy that the mammalian CB1 receptor has been shown to form heteromers with 5-HT2A receptors (Vinals *et al.* 2015).

Although we have yet to identify the molecular target of AM251 in the worm, our data support a model in which AM251 acts upstream of SER-5 and requires TGF-β and insulin peptides in the ASI neuron to promote reproductive growth programs throughout the whole animal. By screening a subset of nematode G-protein mutants, we found that *gpa-3* is required for the effects of AM251, supporting the idea that a GPCR signaling is being targeted. Kim *et al.* (2009) have shown that *gpa-3* mutants are insensitive to ascaroside-induced dauer entry, perhaps suggesting that AM251 is antagonizing a component of the system that is involved in dauer formation in response to ascarosides (Kim *et al.* 2009). However, the CB receptor agonist O-2545, which inhibited the effects of AM251 and also promoted dauer formation when administered alone, was still capable of inducing dauer entry in the absence of *srbc-64* and *srbc-66*, two GPCRs that have been shown to mediate the effects of ascarosides on dauer formation in a *gpa-3* dependent manner (Kim *et al.* 2009). Although other ascaroside receptors exist, these data suggest that synthetic CBs are probably not acting directly on dauer pheromone signaling pathways to modify the dauer decision.

AM251 was developed as a specific antagonist of the mammalian CB1 receptor, but it has since become clear that synthetic CBs are somewhat promiscuous in terms of the receptor subtypes through which they mediate their biological effects (Pertwee *et al.* 2010). As such, AM251 has also been shown to act on multiple targets, including GPR55, T-type calcium channels, voltage gated sodium channels, as well as PPAR α and γ (Pertwee *et al.* 2010). Thus, it is perhaps not surprising to find that AM251 has potent biological effects in an organism that lacks canonical CB receptors (McPartland *et al.* 2006), but is replete with other possible receptor targets that have human homologs (Shaye and Greenwald 2011). However, it is intriguing that other, structurally unrelated CB receptor antagonists also suppressed dauer entry, while CB receptor agonists not only inhibited the effects of AM251, but one of them was able to elicit the opposite phenotype and promote dauer entry when administered alone. Of the antagonists, the CB1 specific compounds, AM251 (Lan *et al.* 1999) and LH21 (Jagerovic *et al.* 2004), were most potent, followed by the selective CB2 antagonist AM630 (Ross *et al.* 1999), while the mixed CB1 antagonist/CB2 agonist, URB447 (LoVerme *et al.* 2009), showed the weakest effects. Of the nonselective CB1/2 agonists CP55,940 (Pertwee 1999) had no effect, while O-2545 (Martin *et al.* 2006) showed the greatest potency, and the CB2 receptor agonist GP1a (Murineddu *et al.* 2006) exhibited a low level of inhibitory activity against AM251. A caveat to interpreting these data are the fact that certain chemical structures accumulate more effectively in the worm than others (Burns *et al.* 2010), and thus those compounds that show weak effects in the worm may simply be those that are taken up poorly. However, taken together, these observations suggest that, if the CB agonists and antagonists are binding to a common receptor in worms, it appears to share some of the pharmacology of both CB1 and CB2 receptors.

In conclusion, we performed a chemical screen for bioactive lipids that modify the dauer phenotype in *C. elegans*, and identified a CB receptor antagonist as a potent suppressor of dauer formation. These studies establish the nematode as a genetically tractable model system that is responsive to synthetic CBs, and therefore could be useful in delineating the classical and nonclassical actions of CB-based therapeutics. The fact that a CB1 receptor agonist and antagonist have opposing effects on the same phenotype in *C. elegans* makes it tempting to speculate that they are acting at a common target. In this respect, there is evidence for the existence of CB receptors in mammals that are distinct from the canonical CB1 and CB2 receptors (Pertwee *et al.* 2010, Brown 2007). Ultimately, studies aimed at identifying the molecular targets of AM251 and O-2545 in *C. elegans* will be required to determine whether the effects of these molecules are mediated via non-CB receptor mechanisms, or via an as yet unidentified noncanonical CB receptor.

## Supplementary Material

Supplemental Material
