# Supplementary material for: Synthetic Ligands of Cannabinoid Receptors Affect Dauer Formation in the Nematode Caenorhabditis elegans
Source: G3 (Bethesda). 2016 Apr 13;6(6):1695–705. doi: 10.1534/g3.116.026997 (PMC4889665; doi:10.1534/g3.116.026997)
Supplement: Supplemental Material [file supp_6_6_1695__index.html]

Synthetic Ligands of Cannabinoid Receptors Affect Dauer Formation in the Nematode Caenorhabditis elegans — Supplemental Material 

# Synthetic Ligands of Cannabinoid Receptors Affect Dauer Formation in the Nematode *Caenorhabditis elegans*

## Supplemental Material for Reis-Rodrigues *et al.*, 2016

**Files in this Data Supplement:**

- File S1 - Figure data. (.xls, 183 KB)
